# Review of a sample of episodes of forced medication in an area of southern Spain

**DOI:** 10.1192/j.eurpsy.2023.2265

**Published:** 2023-07-19

**Authors:** L. I. Muñoz-Manchado, J. M. Mongil-Sanjuan, J. I. Pérez-Revuelta, C. M. Robledo Casal, J. M. Villagrán-Moreno

**Affiliations:** 1 Mental Health Unit; 2 UGC North of Cadiz, Mental Health Inpatient Unit, General Hospital, Jerez de la Frontera. Fundación Biomédica de Cádiz., Jerez de la Frontera, Cádiz., Spain

## Abstract

**Introduction:**

Forced medication is one of the most frequently used coercive measures in acute mental health units. It is a practice that can lead to physical, psychological and psychopathological consequences. Therefore, it is necessary to implement measures to reduce its use. In this sense, it is interesting to study the variables that can be associated with its use, and thus take measures accordingly.

**Objectives:**

This study attempts to identify the number of forced medication episodes between July 2017 and December 2018 treated in the catchment area of the Mental Health Service at Jerez Hospital. As a secondary objective, it pursues to identify the factors that conducted to the use of forced medication with the intention of being able to reduce the use of these measures.

**Methods:**

A descriptive and retrospective study has been developed reviewing the total number of episodes of forced medication. Patients admitted and discharged from hospital between July 2017 and December 2018 treated in the Mental Health Service at Jerez Hospital. Data were extracted from medical records.

**Results:**

The total number of episodes of forced medication identified was 330. In these episodes, the average age was 41 years, with a predominance of 74% of the male gender. The most used route in the episodes was intramuscular (94.8%), in addition, more than 50% needed the association of two drugs, the most used were haloperidol and olanzapine. The 32.7% of the episodes also required the use of mechanical restraint and 44.2% required the presence of security service.

**Image:**

**Figure:**
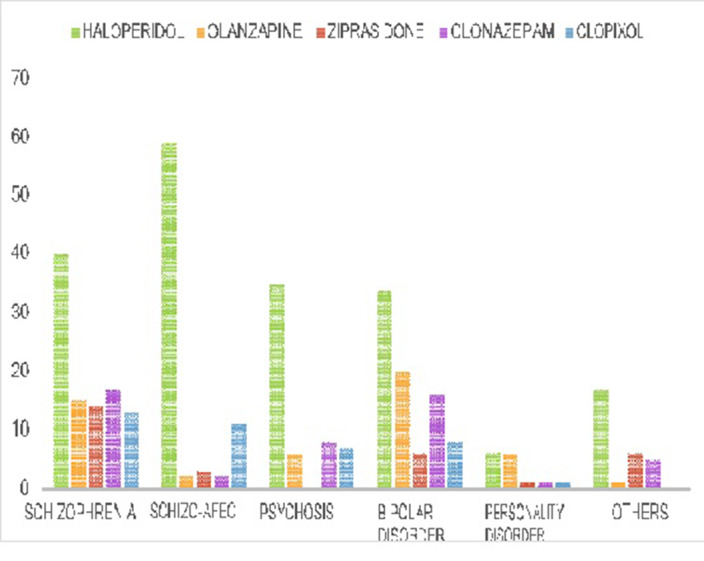

**Conclusions:**

We present the descriptive analysis of a further study currently been conducted in hour hospital which means to stablish predictive factors for the use of forced medication. We therefore intend to create patient profile, as well as new measures specifically directed to these factors with which to diminish the use of forced medication.

**Disclosure of Interest:**

None Declared

